# Isolated Transitory Radial Nerve Palsy as a Complication After Luxatio Erecta Humeri: A Case Report

**DOI:** 10.7759/cureus.69210

**Published:** 2024-09-11

**Authors:** Adnane Lachkar, Najib Abdeljaouad, Hicham Yacoubi

**Affiliations:** 1 Orthopedic Trauma Department B, Mohammed VI University Hospital, Oujda, MAR; 2 Orthopedic Trauma Department B, Faculty of Medicine and Pharmacy of Oujda, Mohamed First University, Oujda, MAR; 3 Orthopedic Department, Mohammed VI University Hospital, Oujda, MAR; 4 Orthopedic Department, Faculty of Medicine and Pharmacy of Oujda, Mohamed First University, Oujda, MAR

**Keywords:** inferior shoulder dislocation, luxatio erecta humeri, radial nerve, radial nerve palsy, scapula and shoulder trauma

## Abstract

Shoulder dislocations are common, with luxatio erecta humeri (LEH) being a rare variant. This report discusses a case of isolated transitory radial nerve palsy following LEH, which occurred after a high-energy motorcycle accident. Neurological examination revealed radial nerve involvement. Following reduction of the dislocation and appropriate physiotherapy, the patient experienced full functional recovery. This case underscores the rarity of isolated radial nerve palsy in LEH and highlights the importance of thorough neurological assessment in such injuries.

## Introduction

Shoulder dislocations are highly prevalent, accounting for approximately 50% of all joint dislocations [1]. Among these, luxatio erecta humeri (LEH) is a notably rare variant, representing less than 1% of shoulder dislocations [2]. The incidence of brachial plexus injuries associated with acute anterior shoulder dislocations is consistently notable [2]. While isolated axillary nerve injuries are commonly observed due to their proximity to the glenohumeral joint [3], isolated radial nerve palsy following anterior shoulder dislocations is less frequently described [4]. The occurrence of isolated radial nerve palsy following LEH is even rarer. This case report presents an unusual instance of isolated transitory radial nerve palsy as a complication following LEH, emphasizing the need for thorough neurological evaluation and the potential for full functional recovery with appropriate treatment and rehabilitation.

## Case presentation

**Figure 1:**
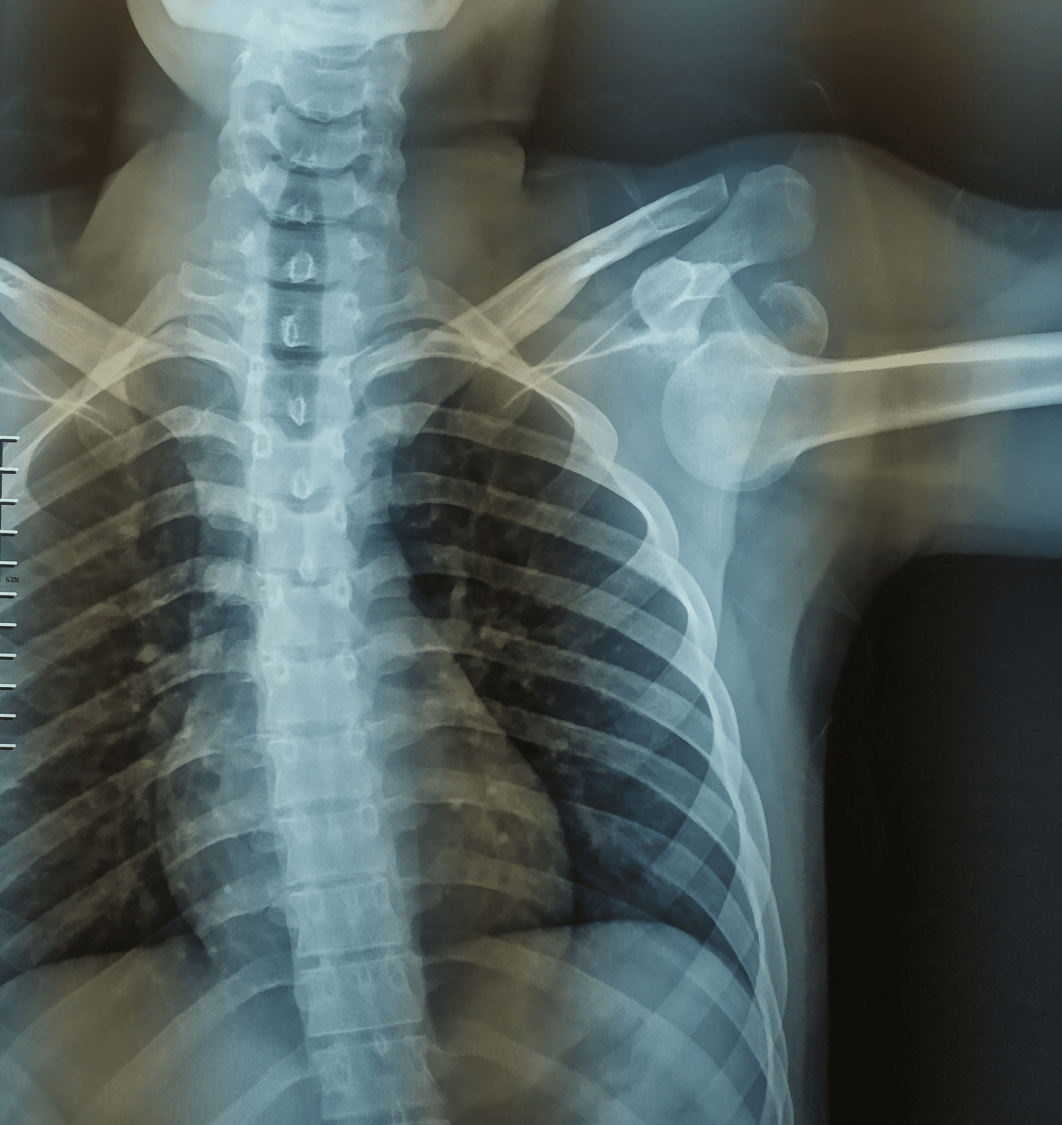
Anteroposterior X-ray of the shoulder showing luxatio erecta with the humeral head displaced inferiorly to the glenoid.

**Figure 2:**
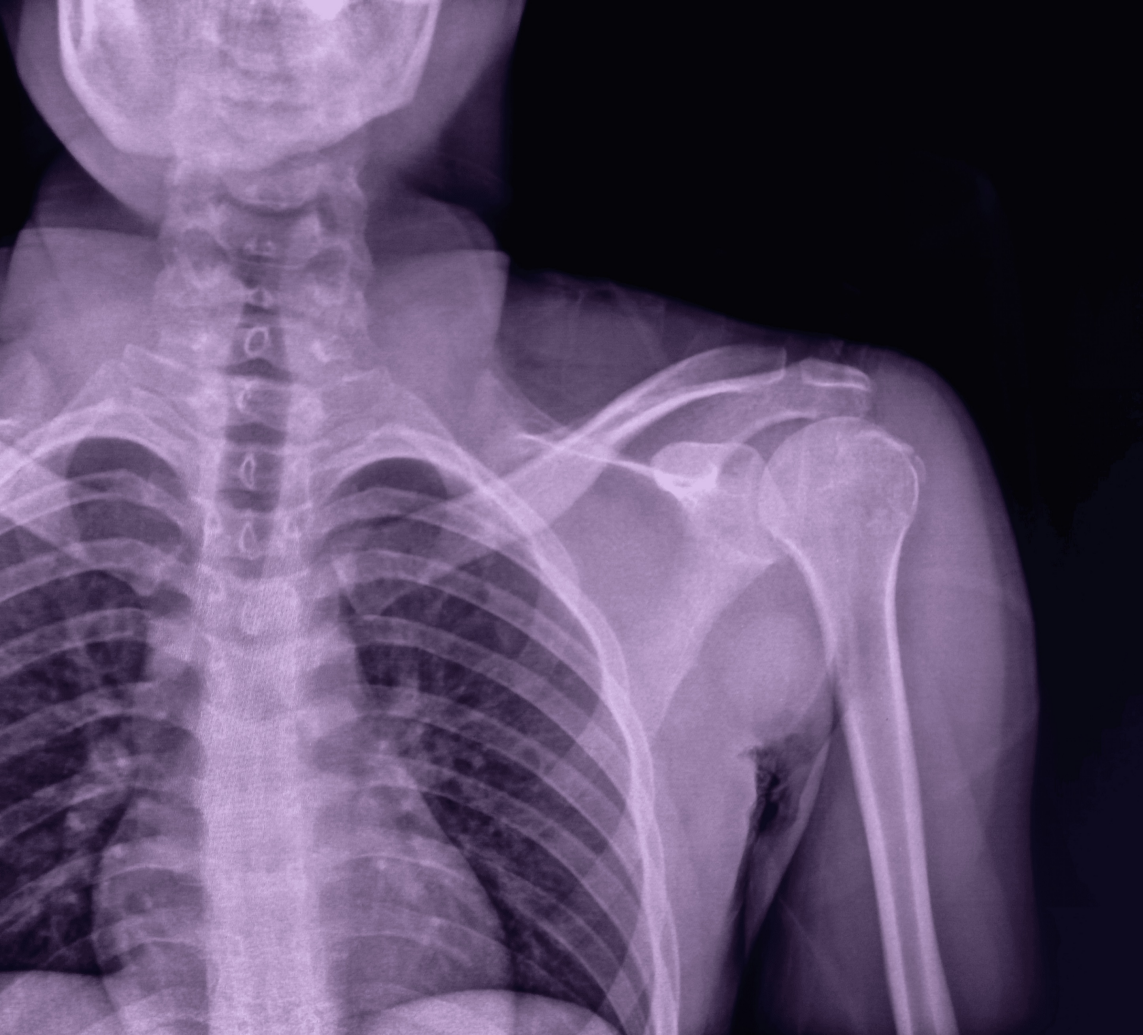
Post-reduction control X-ray showing the correct positioning of the humeral head relative to the glenoid cavity

**Figure 3:**
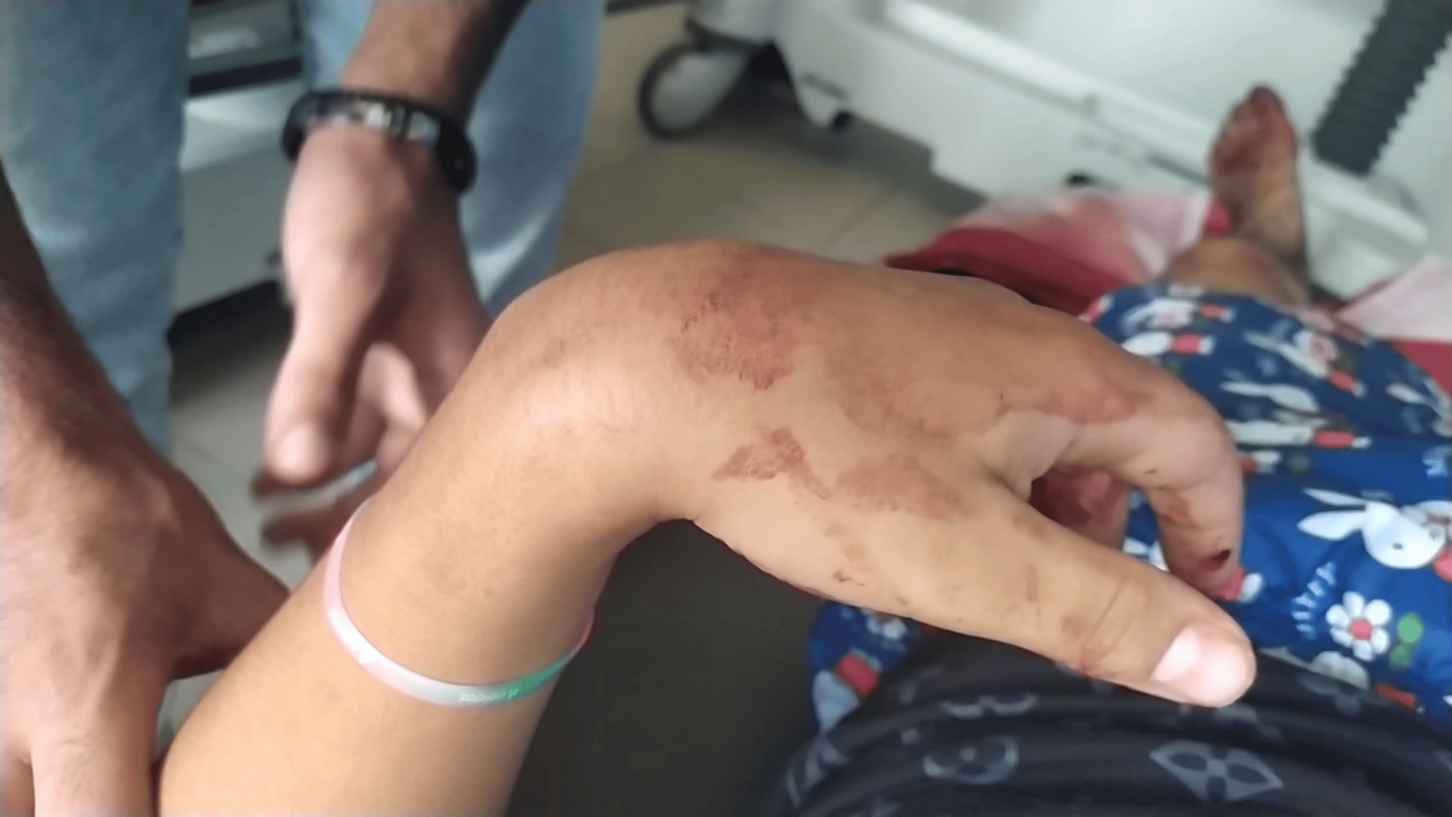
Persistent wrist drop following closed reduction of the dislocation, indicative of radial nerve palsy

A 23-year-old male presented to the emergency department one hour after a motorcycle collision with a tree. He reported severe pain, noticeable bruising, and an inability to move his arm. Physical examination revealed the arm in an abducted position above the head with the elbow flexed at 90 degrees. The patient was unable to lower his arm, and the humeral head was palpable in the axilla. Neurological examination showed numbness along the radial side of the forearm, indicating radial nerve involvement, while the median and ulnar nerves were unaffected. Peripheral pulses (humeral, radial, and ulnar) were weak. An anteroposterior X-ray confirmed inferior shoulder dislocation (LEH) (Figure 1). The dislocation was reduced under sedation using closed reduction via traction-countertraction. Immediately after reduction, the patient received an intravenous bolus of methylprednisolone 125 mg to reduce inflammation and potential nerve compression. Post-reduction X-rays confirmed correct humeral head positioning in the glenoid (Figure 2), but sensory paresthesia and wrist drop persisted (Figure 3). Peripheral pulses normalized and the axillary nerve remained intact. The shoulder was immobilized with a sling, and a wrist splint was applied. The patient was administered ibuprofen 400 mg three times daily and paracetamol 1g every six hours for pain management. Additionally, oral benfotiamine 300 mg daily was prescribed to support nerve recovery.

**Figure 4:**
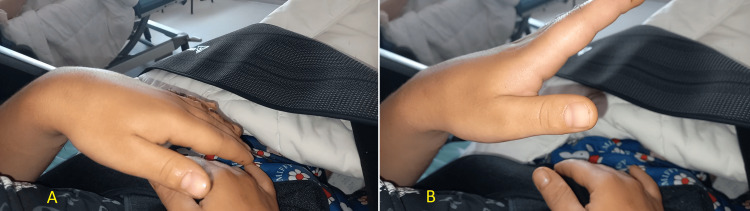
Physical examination one week after the trauma The patient demonstrated slight spontaneous extension of the hand and thumb. A: wrist flexion. B: wrist extension

**Figure 5:**
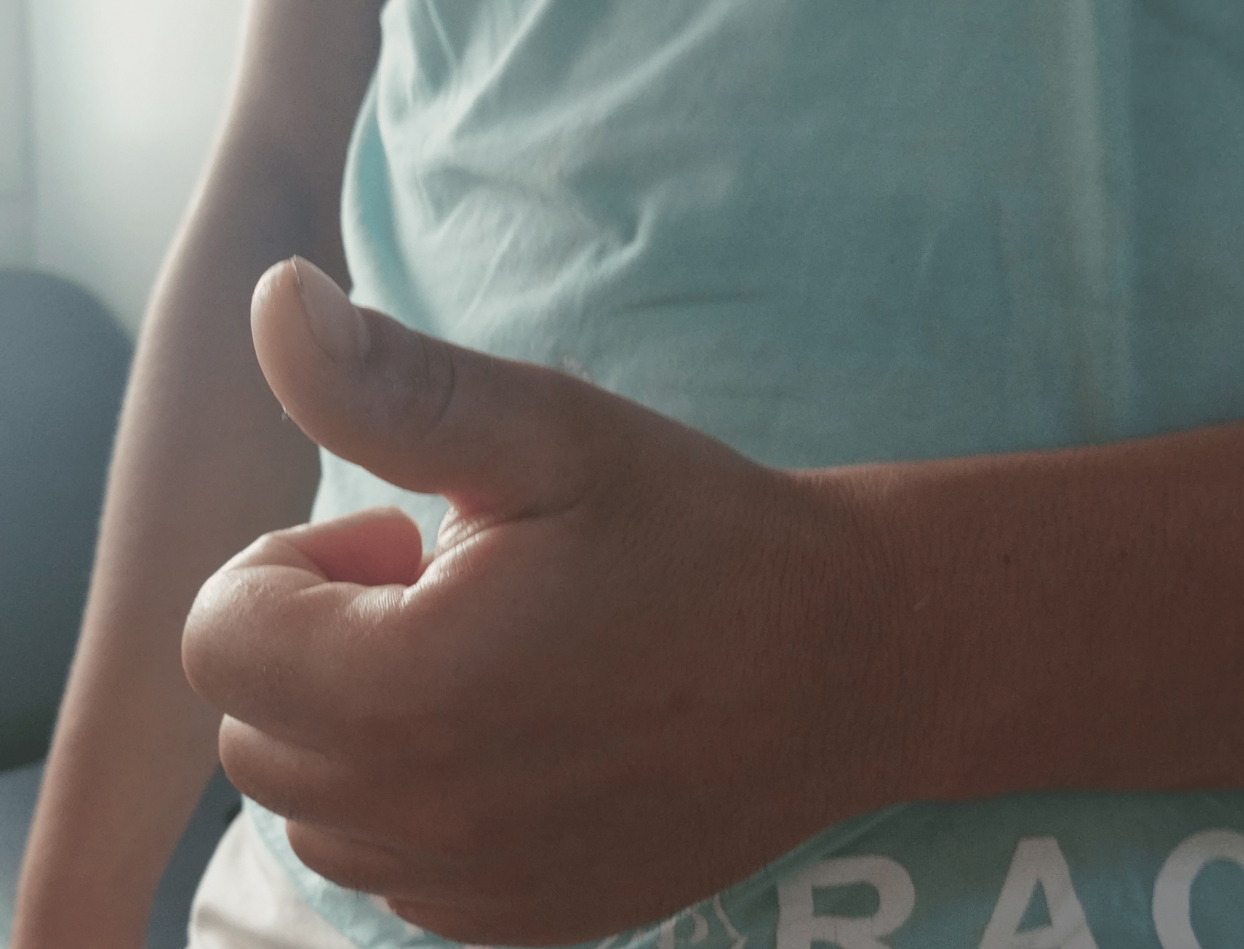
Recovery of thumb extension after four weeks

One day post-trauma, the patient began to regain sensation in the radial nerve distribution. Physiotherapy was initiated to maintain a passive range of motion. By one week, the patient exhibited slight spontaneous extension of the hand and thumb (Figure 4). At this time, he also discontinued all medications, including corticosteroids, analgesics, and benfotiamine. Physiotherapy continued with exercises using resistance devices. Four weeks later, the patient achieved full functional recovery of the arm, with complete restoration of wrist and thumb extension (Figure 5), scoring 5/5 on strength testing. Due to the patient's normal clinical neurological findings, electromyography (EMG) was not performed.

## Discussion

The glenohumeral joint is the most commonly dislocated joint [1] due to its large mobility. Anterior dislocations are the most frequent, whereas LEH, characterized by inferior shoulder dislocation, occurs much less frequently [5]. The term "luxatio erecta" in Latin describes the typical presentation of the arm fully abducted above the head. Such dislocations often result from high-energy trauma, particularly in young males, and frequently involve moderate to severe soft tissue damage [6].

Isolated radial nerve palsies are rare complications of glenohumeral dislocations and are primarily associated with anterior dislocations. Literature suggests that over 10% of dislocations are accompanied by persistent neurological deficits, with the axillary nerve most commonly affected (approximately two-thirds) and the radial nerve least affected (around 1%) [7]. LEH typically occurs due to high-energy trauma, such as falls or vehicle accidents, causing hyperabduction of the humeral neck against the acromion, which displaces the humeral head and tears the inferior capsule [6]. This high-energy impact can damage nerves due to excessive traction and the short anchorage points of nerves in the upper limb. The radial nerve, originating from the posterior cord of the infraclavicular brachial plexus, innervates the extensor muscles of the forearm and arm. The inferior displacement of the humeral head in luxatio erecta is likely the primary cause of nerve traction and resultant radial nerve palsy.

In a study by Ostermann et al. [6], 21% of patients experienced neurologic injuries following an anteroinferior shoulder dislocation (LEH). This increased rate of neurologic complications compared to those observed with other shoulder dislocations may be linked to the anatomical proximity of the brachial plexus, particularly the axillary nerve, to the inferior glenoid rim. Although this hypothesis cannot be definitively confirmed in the current case report, it is plausible that the brachial plexus is at a higher risk of injury when the humeral head is dislocated beneath the inferior glenoid rim. Various studies have indicated that vascular or nerve injuries can occur at the time of dislocation or during the reduction process [8, 9]. Consequently, a comprehensive neurological examination before and after reduction is crucial. Without a pre-manipulation neurological assessment, nerve deficits may be incorrectly attributed to iatrogenic injury. Typically, neurological dysfunction resolves with shoulder reduction, as brachial plexopathy decreases [6]. Nerve injuries resulting from shoulder dislocation are generally neuropraxic or axonotmetic, with favorable recovery outcomes [2, 4]. Neuropraxia often shows near-complete recovery within three months, whereas axonotmetic injuries usually demonstrate reinnervation and recovery within six to seven months [10]. EMG studies are recommended three weeks post-injury for persistent upper limb palsies or paralysis. If recovery does not occur, nerve transfers may be considered [11].

## Conclusions

Isolated radial nerve palsy following LEH is exceptionally rare, with this case report providing valuable insight into its occurrence and management. The patient’s full recovery underscores the potential for positive outcomes with appropriate treatment and rehabilitation. This case emphasizes the necessity of early and accurate neurological evaluation in shoulder dislocations to avoid misattribution of nerve deficits to iatrogenic causes and to ensure optimal recovery. Further studies are needed to better understand the mechanisms of nerve injury in luxatio erecta and to refine management strategies for similar cases.
